# Predicting later categories of upper limb activity from earlier clinical assessments following stroke: an exploratory analysis

**DOI:** 10.1186/s12984-023-01148-1

**Published:** 2023-02-21

**Authors:** Jessica Barth, Keith R. Lohse, Marghuretta D. Bland, Catherine E. Lang

**Affiliations:** 1 Program in Physical Therapy, Washington University School of Medicine, St. Louis, MO USA; 2 Program in Occupational Therapy, Washington University School of Medicine, St. Louis, MO USA; 3 Department of Neurology, Washington University School of Medicine, St. Louis, MO USA

**Keywords:** Upper extremity, Accelerometry, Supervised machine learning, Rehabilitation, Outcome assessments, Stroke

## Abstract

**Background:**

Accelerometers allow for direct measurement of upper limb (UL) activity. Recently, multi-dimensional categories of UL performance have been formed to provide a more complete measure of UL use in daily life. Prediction of motor outcomes after stroke have tremendous clinical utility and a next step is to explore what factors might predict someone’s subsequent UL performance category.

**Purpose:**

To explore how different machine learning techniques can be used to understand how clinical measures and participant demographics captured early after stroke are associated with the subsequent UL performance categories.

**Methods:**

This study analyzed data from two time points from a previous cohort (n = 54). Data used was participant characteristics and clinical measures from early after stroke and a previously established category of UL performance at a later post stroke time point. Different machine learning techniques (a single decision tree, bagged trees, and random forests) were used to build predictive models with different input variables. Model performance was quantified with the explanatory power (in-sample accuracy), predictive power (out-of-bag estimate of error), and variable importance.

**Results:**

A total of seven models were built, including one single decision tree, three bagged trees, and three random forests. Measures of UL impairment and capacity were the most important predictors of the subsequent UL performance category, regardless of the machine learning algorithm used. Other non-motor clinical measures emerged as key predictors, while participant demographics predictors (with the exception of age) were generally less important across the models. Models built with the bagging algorithms outperformed the single decision tree for in-sample accuracy (26–30% better classification) but had only modest cross-validation accuracy (48–55% out of bag classification).

**Conclusions:**

UL clinical measures were the most important predictors of the subsequent UL performance category in this exploratory analysis regardless of the machine learning algorithm used. Interestingly, cognitive and affective measures emerged as important predictors when the number of input variables was expanded. These results reinforce that UL performance, in vivo, is not a simple product of body functions nor the capacity for movement, instead being a complex phenomenon dependent on many physiological and psychological factors. Utilizing machine learning, this exploratory analysis is a productive step toward the prediction of UL performance.

*Trial registration* NA

**Supplementary Information:**

The online version contains supplementary material available at 10.1186/s12984-023-01148-1.

## Background

Wearable movement sensors allow for direct measurement of upper limb (UL) activity in daily life, i.e. performance [1]. Performance is operationally defined in the World Health Organization’s (WHO) International Classification of Function (ICF) model as activity in the unstructured, free-living environment, and is distinguished from capacity, operationally defined as the capability for activity in a structured or standardized environment [2, 3]. The most common wearable sensors used are accelerometers, from which numerous clinically relevant variables about UL activity can be computed to provide insight into how people with or without neurological impairment use their ULs in daily life [4–7]. Data extracted from bilateral, wrist-worn wearable sensors can be used to quantify UL performance variables measuring the duration [8, 9], symmetry [6, 8, 10, 11], magnitude [5, 7, 12], and variability of one or both limbs [5, 7, 12]. Each UL performance variable conveys slightly different information about the collective nature of UL use; multiple variables may provide a fuller understanding of the scope of UL performance in daily life [13]. As a solution to the multi-variable problem, we recently categorized UL performance in adult cohorts with and without stroke [14]. The most parsimonious solution was five categories of UL performance formed from five UL performance variables named: (A) Minimal activity/rare integration; (B) Minimal activity/limited integration; (C) Moderate activity/moderate integration; (D) Moderate activity/full integration; (E) High activity/full integration. The UL performance categories are multi-dimensional, with each category providing information about UL activity with respect to the different movement characteristics in adults with and without neurological UL deficits. Thus, the five categories of UL performance may provide a more complete measure of UL use in daily life [13, 14].

Early prediction of motor outcomes after stroke has tremendous clinical utility [15, 16]. Our next step, therefore, was to explore what factors might predict someone’s subsequent UL performance category. Predictive knowledge of subsequent outcomes can inform the delivery and specification of individualized rehabilitation services [17, 18]. This effort to predict an individual’s subsequent UL performance category is informed by the development of the PREP 2 algorithm [19, 20], which has demonstrated that prediction of an UL capacity (i.e. activity a person has the capability to do) category provides clinically-useful information to people with stroke and their families [21–24]. Advances in computing have improved upon old and led to new analysis techniques for building prediction models of UL outcomes after stroke. Recently, machine learning techniques of support vector machines (SVM) and tree-based methods (e.g., Classification and Regression Trees [CARTs]) have been used to classify people with stroke into categories with different ranges of UL capacity [17, 20, 25–27]. The PREP 2 prediction model was originally built and validated with a CART which resulted in the easy to interpret decision tree [20]. Machine learning techniques have the advantages of: (1) requiring fewer assumptions about the distributions of the data, (2) numerous options for non-parametric models, and (3) strong predictive capabilities [18, 26–29]. There are strengths and weaknesses to each machine learning technique. For example, the CART algorithm yields a single, easy to interpret decision tree (strength), but lower predictive accuracy on new, external samples because of high variance (weakness) [30]. An alternative to creating a single decision tree is to use ensemble classifiers like *b*ootstrap *agg*regation (called “bagging”) or random forests [31]. These ensemble techniques rely on the collective judgment of many decision trees (hundreds or even thousands) in order to make a classification. These ensemble methods tend to have higher predictive power and reduce the risk of over-fitting relative to other CART methods, but at the expense of interpretability (as there is no longer one single decision tree to follow, but a whole forest of trees) [26, 31]. Capitalizing on the advantages of ensemble machine learning algorithms by applying them for prediction of UL performance outcomes could yield key insights into UL recovery post stroke.

The purpose of this study, therefore, was to explore how different machine learning techniques can be used to understand how clinical measures and participant demographics captured early after stroke are associated with the UL performance categories from a later post stroke time point. We utilized the same data set from which we had previously predicted the trajectory of single, continuous UL performance variables with regression techniques [32]. In this analysis, we attempt to predict the subsequent multivariate categories of UL performance that people with stroke fell into. We explicitly tested different machine learning methods to build predictive models with different input variables as predictors (also called feature sets) to explore how each method yields similar versus different results. Based on prior post stroke prediction models of UL capacity [17, 19, 33], performance [25, 34], and walking performance [18, 35–37], we hypothesized that the Shoulder Abduction Finger Extension (SAFE) measure of UL impairment [20], the Action Research Arm Test (ARAT) a measure of UL capacity [17, 32], the Area Deprivation Index (ADI) [38–40] and the Center for Epidemiological Studies Depression Scale (CES-D) would be the most important predictors of the subsequent UL performance category.

## Methods

This study was a secondary analysis of data collected from a prospective, observational, longitudinal cohort, tracking UL change over time [32]. Sources of data from two time points were participant characteristics, clinical measures from early after stroke (within two weeks of onset), and subsequent categories of UL performance (from a previous report) [14] later after stroke.

### Participants

Participants were included in the prospective, observational, longitudinal cohort if the following criteria were met: (1) within two weeks of first-ever ischemic or hemorrhagic stroke, confirmed with neuroimaging; (2) presence of UL motor deficits within the first 24–48 h post stroke, as indicated by a NIHSS [41]. Arm Item scores of one to four or documented manual muscle test grade [42] of < 5 anywhere on the paretic UL; (3) ability to follow a two-step command, as measured by a NIHSS [41]. Command Items score of zero; and (4) anticipated return to independent living (i.e., not institutionalized), as indicated by the acute stroke team. Persons with stroke were excluded if any of the following criteria were met: (1) history of previous stroke, other neurologic condition, or psychiatric diagnoses; (2) presence of comorbid conditions that may limit recovery (e.g., end-stage renal disease or stage IV cancer); (3) lived more than 90 min from the study location; and (4) currently pregnant by self-report. The Human Research Protection Office at Washington University in St. Louis approved this study, and all participants provided written informed consent.

### Data collection

Cohort participants completed eight assessment sessions over the first 24 weeks post stroke. This analysis used data from the first assessment (within two weeks of stroke onset) to predict the subsequent category of UL performance (14) from the latest time point between six and 24 weeks post stroke. We retained any person in the cohort whose last measurement was between six and 24 weeks post stroke because UL performance appears to stabilize between three and six weeks [9, 32]. Participants were excluded from this analysis if they were missing any of the predictor variables from the first assessment point (see Table 1). Assessments were administered by trained personnel (licensed physical therapists or occupational therapists, range of experience with measures was 2–15 years). Since this was an observational cohort study, we did not provide nor control for the amount or type of rehabilitation services delivered to enrolled participants. Participants received rehabilitation services as prescribed by their medical team.

**Table 1:** Predictors included in the analysis

Predictor name	Description	Construct	Scoring
UL measures			
Action Research Arm Test (ARAT) [45–47]	Standardized measure assessing UL functional ability for activity	UL capacity	Scores range from 0–57, higher values indicate greater UL function
Shoulder Abduction Finger Extension (SAFE) [20, 23]	Sum of two Medical Research Council strength grades from the shoulder abductors and the finger extensors	UL impairment	Scores are whole numbers and range from 0–10, higher values indicate less impairment in the affected UE
Upper Extremity Fugl-Meyer (UEFM) [48, 49]	Standardized measure assessing movement in and out of synergies of the affected UL	UL impairment	Scores range from 0–66, higher values indicate less impairment in the affected UL
Non-motor clinical measures			
Center for Epidemiological Studies Depression Scale (CES-D) [50, 51]	Questionnaire asking about the frequency and severity of symptoms associated with mood	Depression screen	Scores range from 0–60, higher scores indicative of greater depressive symptomatology
Unstructured Mesulam [52, 53]	Paper, pencil test measuring visual spatial ability	Hemispatial neglect	Scores are calculated by subtracting the omissions from the total score of 60 (0–30 on both sides), > 4 omissions on one side are considered pathological
Montreal Cognitive Assessment (MOCA) [54]	Brief tool to screen for cognitive impairment across multiple domains	Cognitive screen	Scores range from 0–30, scores < 26 indicate cognitive impairment
National Institute of Health Stroke Scale (NIHSS) [55]	Standardized measure of global stroke severity	Stroke severity	Scores range from 0–42, lower scores indicate less severe stroke overall
Demographics			
Area deprivation index (ADI) [38–40]	Multi-dimensional evaluation of a region’s socioeconomic conditions	Socioeconomic disadvantage	Scores are % rankings and range from 1 to 100, lower values indicate lowest level of “disadvantage”
Age	Participant age at time of testing	–	Minimum age of 18
Concordance	Affected UL is dominant UL	–	Categorized as: Yes/No
Ethnicity	Participant report of ethnicity	–	Categorized as: Non-Hispanic/Non-Latino or Latino
Living status pre-stroke	Participant report of prior living situation	–	Categorized as*: Living alone, independent ADLs/Living alone, assist ADLs/ Living with others, independent ADLs/ Living with others, assist ADLs
Living status 2-weeks post stroke	Participant location at 2-week time point	–	Categorized as: Inpatient/Skilled-nursing facility/Assisted living/Home/Other
Race	Participant reported racial identification	–	Categorized as: White/ Black or African-American/Asian/American Indian or Alaska Native/Hawaiian or other Pacific Islander
Sex	Participant report of sex	–	Categorized as: Male/Female
Stroke type	Cause of disruption of blood flow, from medical record	–	Categorized as: Ischemic/Hemorrhagic/Unknown
Time post stroke	Number of days from stroke-onset to 2-week testing, from medical record	–	Difference in days between date of testing and stroke onset

* Activities of daily living (ADL)

### Dependent variable used for the models

**Fig. 1:**
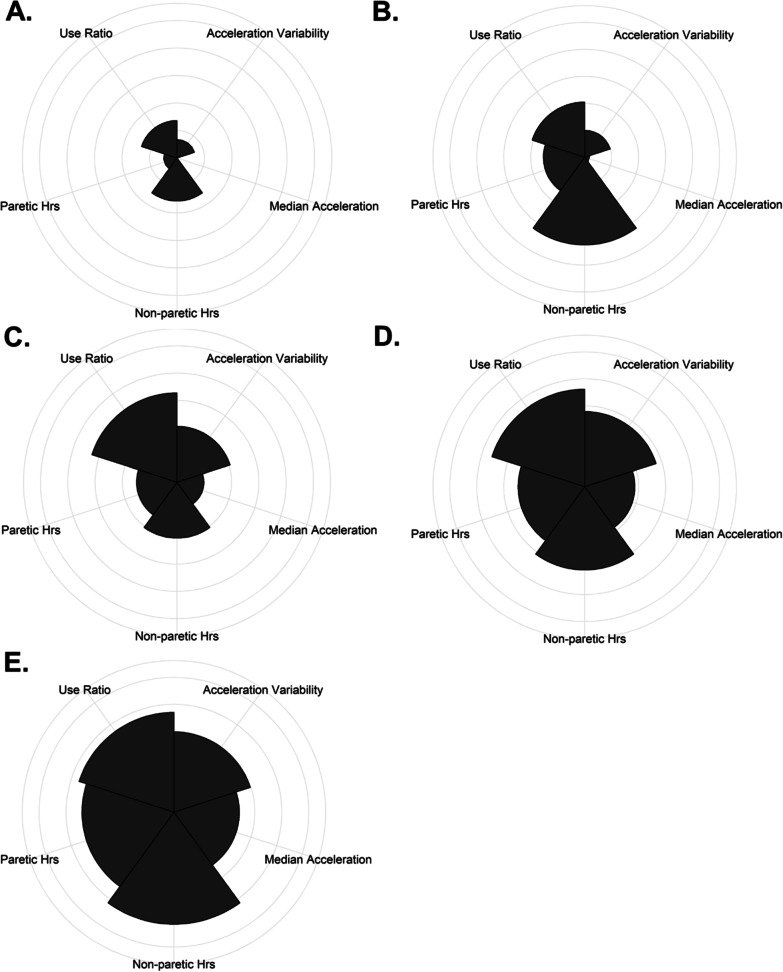
Coxcomb charts of the five UL performance categories of the 54 participants in this analysis (categories assigned in Barth et.al 2021). The five UL performance variables are divided into equally segmented wedges on the radial chart and the area of each wedge is proportional to the magnitude of the score on that dimension relative to the sample that created the categories. Each chart illustrates the contribution of the five UL performance variables on a standardized scale and are anchored to the minimum and maximum value of each variable in the prior analysis used to establish the categories. The categories are presented in order of increasing overall UL performance and are named: **A** minimal activity/rare integration; **B** minimal activity/limited integration; **C** moderate activity/moderate integration; **D** moderate activity/full integration; **E** high activity/full integration

The dependent variable (outcome or class in machine learning) in this analysis was a category of UL performance established in previous report [14]. These were derived from UL performance variables quantified via accelerometer data [14]. Participants in the prospective, longitudinal, observational, cohort wore Actigraph GT9X-Link accelerometers on both wrists at each time point with methods previously described [1]. Briefly, tri-axial acceleration data are sampled at 30 Hz for 24 or more hours continuously. Once the accelerometers were returned to the lab, data were uploaded, visually inspected, and processed using ActiLife 6 (Actigraph Corp., Pensacola, FL) proprietary software. No activities (e.g. walking or sleep) were excluded from the recordings. For most variables, data were band-pass filtered (0.25–2.5 Hz) and down sampled into one-second epochs with ActiLife proprietary software, where each second is the sum of the 30 Hz values in that second and converted into activity counts (unit of acceleration recorded for this device and software, 1 count = 0.001664 g). Similar to previous work [7–9, 32], accelerometry data was processed using custom written software in MATLAB (Mathworks, Inc., Natick, MA) to calculate UL performance variables which quantify various aspects of UL activity in everyday life. The variables measure the duration, magnitude, variability, symmetry, and quality of movement of one or both ULs. A total of twelve cluster solutions (3-, 4-, or 5- clusters based on 12, 9, 7, or 5 input variables) were calculated to systematically evaluate the most parsimonious solution. Quality metrics and principal component analysis of each solution were calculated to arrive at a locally-optimal solution with respect to the number of input variables and the number of clusters. Across different numbers of input variables, two principal components consistently explained the most variance. Across the models with differing numbers of UL input performance variables, a five-cluster solution formed from five UL performance variables explained the most overall total variance (79%) and had the best model-fit. The five performance variables selected measure the duration (hours of use of the paretic and non-paretic limb), symmetry (use ratio), variability (acceleration variability of the paretic UL), and magnitude (median acceleration of the paretic UL) of UL activity. The participants’ category assignment in the prior report was the categorical outcome in this analysis [14]. The names of each of the five categories were chosen for their overall level of UL activity and the integration of both ULs into activity in daily life and are named: (A) Minimal activity/rare integration; (B) Minimal activity/limited integration; (C) Moderate activity/moderate integration; (D) Moderate activity/full integration; and (E) High activity/full integration, see Fig. 1 for a visual representation of the categories for the participants included in this analyses. The categories are presented in order of increasing overall UL performance [14]. The category with the lowest UL performance is the Minimal activity/rare integration*,* this category has the lowest mean values on variables that quantify duration, magnitude, and variability of UL activity. People in this category use their non-paretic UL approximately 2.5 times more than their paretic UL and have little to no magnitude or variability of their paretic UL activity in daily life. People in the Minimal activity/limited integration category use both the paretic and non-paretic limb for more overall hours than the Minimal activity/rare integration category, but the non-paretic limb is still active twice as much as the paretic UL. Additionally, people in this category have slightly higher mean values on performance variables that quantify both the magnitude and variability of the paretic limb when compared to the Minimal activity/rare integration category. The category with overall, moderate UL performance is the Moderate activity/moderate integration category. In this category, people have more symmetrical UL use compared to the two lower categories and moderate values on variables that quantify both the magnitude and variability of paretic limb activity. The two categories with the highest overall UL performance are the Moderate activity/full integration and the High activity/full integration categories. These categories have progressively higher mean values of variables quantifying the duration, magnitude, and variability of UL activity with those in the High activity/full integration category having the highest mean values compared to the other categories. Both of these categories, however, have similar mean values of the use ratio indicating that people in these two categories have relatively equal contributions of both ULs during daily life [14].

### Independent predictor variables

The input variables (also known as feature sets in machine learning) were participant demographics and clinical measures. In the prospective, longitudinal, observational cohort, 15 demographic variables and nine clinical measures were administered at the first assessment time point, within two weeks post stroke. Of the 24 variables available, seven were excluded because of multi-collinearity and extremely low (or no) variability [43]. The correlations between the continuous variables included in this analysis are presented in Additional file 1: Fig. S1 and the frequencies of the excluded factor variables in Additional file 2: Table S1. In the case of multi-collinearity, we retained the variables that were more likely to be available in routine post stroke clinical care [44]. Table 1 presents the 17 predictors selected for this analysis organized into three main categories: (1) UL clinical measures; (2) non-motor clinical measures; and (3) demographics.

### Statistical analysis

All data were analyzed in R (version 4.1.2), an open source statistical computing program [56]. Distributions and pair-wise scatterplots of the correlations between the variables were examined to understand the variability in the sample and the relationships among the variables. We tested a series of supervised machine learning algorithms with different numbers of input variables to predict subsequent activity of the UL measured via accelerometry as a function of clinical and demographic variables collected within two weeks of stroke onset. These algorithms were a single decision tree [57], bagged trees, and random forests [58]. We present several different measures of classification accuracy and the importance of different predictor variables [59].

#### Classification using supervised learning algorithms

In this analysis, different machine learning techniques were explored to understand how clinical variables captured early after stroke best predicted a subsequent category of UL performance, established in a previous analysis. Given the smaller sample size (n = 54) we lacked the capability to partition the data into training, validation, and testing sets. As such, our focus is on the accuracy of the in-sample prediction (how well the model explains the data on which it was trained), cross-validation accuracy (the out-of-bag error estimate, defined below), and measures of variable importance (to identify the most important predictors) [28, 29]. The models were built with different machine learning algorithms as described in the steps below.

First, a single unpruned classification tree was built [57] using the CART algorithm [30, 57]. If one thinks of the dataset as a matrix, each person (or observation) is a row and each predictor (or feature) is a column. The algorithm looks at all predictors and selects the one that best explains the outcome, creating a “branch” in the growing tree (if the predictor is > a certain value, go left, otherwise, go right). Moving down each branch, the algorithm then looks at all remaining predictors, selecting the one that explains the most variance (i.e., creates the most separation in predictions based on the Gini-index, a measure of node purity) [29, 60]. In our analysis, we built an unpruned single decision tree based on all 17 predictors (Table 1) to predict the UL performance categories (outcomes) [29, 60]. The process then repeats, creating a tree made up of many branches that ends in the “leaves”, the final prediction at the end of that branch [28, 30].

Second, we used *b*ootstrap *agg*regation (*bagging*) as an ensemble method to reduce the likelihood of overfitting the data with a single tree [28]. Bagging works identically to the single tree algorithm above, but rather than building one tree out of all available data, samples are bootstrapped: made by randomly sampling individuals (rows) from the data with replacement. Each sample then gets its own tree based on the individuals who made it “in the *bag*”. Critically, this also means that the accuracy of each individual tree can be cross-validated against the observations left “out-of-bag”, yielding out-of-bag error as measure of cross-validation accuracy [28]. The bagged model is thus the aggregated vote of all of the different trees when given input data for classification.

Finally, we also used random forests as a slightly more complicated ensemble method [28, 60]. The random forest is constructed similarly to the bagged trees, building bootstrapped samples and fitting trees within each sample. However, in order to avoid potential bias and correlations between trees (i.e., a dominant predictor always being selected first), the random forest only considers a random subset of predictors (columns) at each node [60]. Thus, the random forest model allows for a similar calculation of cross-validation accuracy with the out-of-bag error, but generally leads to trees being less similar than in bagging, because only a subset of predictors are considered at each node. This more diverse forest of uncorrelated trees can then be used to get an aggregate vote when given input data for classification [28, 60].

A total of six models were built with the two bagging algorithms and by systematically changing the model specifications, known as tuning parameters. Table 2 presents the model names and specifications for the six models built. Each of the six models were built with the number of trees held constant at 2000. A high number of trees was chosen to ensure that all of the models would stabilize regardless of the data set used [29]. Three different input data sets were formed from the list of predictors (Table 1). The small data set included the UL clinical measures, the medium included the small data set + other non-motor clinical measures, and the large data set included the medium data set + demographic predictors. The bagged and random forest models were built by changing the tuning parameter *m*, which is the number of predictors available at each split. In the bagged models, *m* is equal to the total number of predictors in the data set whereas in random forests, *m* is equal to the square root of the number of predictors in the data set (last column Table 2). The splitting criteria (i.e. cost function) used in the random forest models is the Gini Index [61].

**Table 2:** Model names and specifications of the six ensemble models

Model	# Trees	Predictors Included in Input Data Set			# Predictors considered at each node (*m* =)^*^
		UL clinical measures	Non-motor clinical measures	Demographics	
Small bagged	2000	✔	✗	✗	3
Small random forest	2000	✔	✗	✗	√3 = 2
Medium bagged	2000	✔	✔	✗	7
Medium random forest	2000	✔	✔	✗	√7 = 3
Large bagged	2000	✔	✔	✔	17
Large random forest	2000	✔	✔	✔	√17 = 4

*m is a tuning parameter for the bagged trees and random forest models

#### Model performance and variable importance

An iterative process was used to quantify the explanatory power (in-sample accuracy), predictive power (out-of-bag estimate of error), and variable importance of the single decision tree, bagged models, and random forests. For all seven models, the full data set was fed back into the fitted model and the “in-sample” accuracy was quantified by comparing the predicted category and the actual UL performance categories [28, 60]. As in-sample accuracy uses the same data the model is trained on, it is best thought of as the “explanation” rather than “prediction” (because prediction requires an independent test data set). Second, for the bagged and random forest models the average out-of-bag error was used as a measure of cross-validation accuracy. This out-of-bag error is a genuine prediction because each individual tree is independent of its out-of-bag data [29]. (Note that out-of-bag error cannot be calculated for the single tree, as all data are included in the training set for that tree.) Finally, the importance of each predictor for the six models was evaluated in two ways: (1) mean change in accuracy, and (2) mean change in the Gini index [28, 29]. The mean change in accuracy is the improvement or decrease in the in-sample accuracy when each predictor is included in the model, predictors with higher accuracy values are more important for the successful classification (accuracy) of the outcome. Predictors with negative accuracy values decrease the model performance (accuracy) and are considered unimportant in predicting the outcome. The mean change in the Gini index is a measure of how each predictor contributes to the purity of the nodes and leaves in the models. The mean change in the Gini index is a positive integer, higher values of the mean change in the Gini index indicate greater importance of that predictor for the models.

## Results

Overall, the sample of persons with first-ever stroke were generally in their 60’s and had mild to moderate stroke (83% with NIHSS 0–15). Of the 67 participants enrolled in the prospective, observational, longitudinal cohort study [32], 57 were included in the formation of the UL performance categories and 54 had the necessary data to be included in this secondary analyses. Participant assignment into the five UL performance categories was pulled from our previous report [14]. The early predictors were captured at a median of 13 days post stroke (IQR: 12,15) and 67% of the participants UL performance categories were established from wearable sensor data recorded at the week 24 assessment point. Demographics of the 54 participants and for the subsets assigned to the UL performance categories are provided in Table 3. Values are presented as means and standard deviations for normally distributed variables and medians and first and third quartile values for non-normally distributed values. Figure 1 is included for descriptive purposes as a visual representation of the five UL performance categories. In Fig. 1, categories (1A–1E) are represented by Coxcomb plots, where the individual variables used to define the categories are wedges. As one moves from Fig. 1A–E, the wedges take on different relative proportions, generally getting larger, with the best UL performance represented by category E, High activity/full integration.

**Table 3:** Participant characteristics and demographics: Total sample and UL performance category

Characteristic	Total Sample N = 54	A: Min activity/rare Integration N = 20	B: Min activity/limited integration N = 4	C: Mod activity/moderate integration N = 16	D: Mod activity/full integration N = 10	E: High activity/full integration N = 4
Age	66.3 ± 8.8	69.0 ± 7.8	63.3 ± 8.1	65.4 ± 10.1	65.6 ± 8.6	61.3 ± 10.4
Sex, n (%)						
Male	31 (57)	10 (50)	1 (25)	10 (63)	7 (70)	3 (25)
Female	23 (43)	10 (50)	3 (75)	6 (11)	3 (30)	1 (25)
Ethnicity, n (%)						
Non-Hispanic/Non-Latino	54 (100)	20 (100)	4 (100)	16 (100)	10 (100)	4 (100)
Race, n (%)						
White	32 (59)	12 (60)	4 (100)	6 (38)	7 (70)	3 (75)
African-American	21 (39)	8 (40)	–	9 (56)	3 (30)	1 (25)
Asian	1 (2)	–	–	1 (6)	–	
Stroke type, n (%)						
Ischemic	48 (89)	20 (100)	4 (100)	11(69)	9 (90)	4 (100)
Hemorrhagic	6 (11)	–	–	5 (31)	1 (10)	–
Concordance, n (%)	23 (43)	8 (40)	1 (25)	4 (25)	7 (70)	3 (75)
Time post stroke in days^*^	13 (12,15)	13 (12,14)	14 (13,15)	13 (12,14)	15 (13,16)	16 (13, 18)
Week post stroke of UL performance category, n (%)						
Week 6	2 (4)	1 (5)	–	–	1 (10)	–
Week 8	4 (7)	3 (15)	–	1 (6)	–	–
Week 12	5 (9)	3 (15)	–	2 (13)	–	–
Week 16	4 (7)	2 (10)	–	1 (6)	1 (10)	–
Week 20	3 (6)	2 (10)	–	1 (6)	–	–
Week 24	36 (67)	9 (45)	4 (100)	11 (69)	8 (80)	4 (100)
Living status pre-stroke n (%)						
Alone, independent	11 (20)	5 (25)	1 (25)	5 (31)	–	–
Others, independent	43 (80)	15 (75)	3 (75)	11 (69)	10 (100)	4 (100)
Living status 2-weeks post stroke, n (%)						
Inpatient	47 (87)	20 (100)	3 (75)	14 (87)	7 (70)	3 (75)
Home	7 (13)	–	1 (25)	2 (13)	3 (30)	1 (25)
ADI	75 (39, 86)	80 (41, 88)	66 (48, 78)	76 (45, 87)	67 (31, 84)	31 (21, 47)
Upper limb measures						
ARAT	20 (0, 43)	0 (0, 3.3)	4 (0, 20)	37 (23, 48)	45 (30, 53)	37 (28, 41)
SAFE	7 (1, 8)	1 (1, 4)	5 (1, 8)	8 (7, 8)	8 (8, 8)	8 (7, 8)
UEFM	37 (10, 57)	10 (8, 21.3)	23 (9, 43)	54 (36, 56)	59 (48, 61)	54 (43, 57)
Non-motor clinical measures						
CES-D	14.0 ± 9.5	18.2 ± 10.3	14.0 ± 9.1	15.5 ± 8.2	7.0 ± 6.1	4.8 ± 1.7
Mesulam	0 (0, 3)	1 (0, 7)	1 (0, 4)	1 (0, 4)	0 (0, 1)	-1 (-1, 1)
MOCA	17.6 ± 7.1	14.6 ± 7.9	20.3 ± 8.3	18.3 ± 7.1	20.5 ± 4.1	20.3 ± 4.4
NIHSS	6 (4, 10)	10 (6, 15)	5 (4, 9)	5 (3, 8)	4 (4, 6)	3 (3, 4)

*Time post stroke indicates when, in days, the predictor variables were measured

Summary statistics for demographic information and the predictors as means ± standard deviations when normally distributed, otherwise by medians and the 1st and 3rd inter-quartile values in parentheses. Categorical variables are presented as count (%) of the total sample and by category

ADI: Area deprivation index; ARAT: Action Research Arm Test; SAFE: Shoulder Abduction Finger Extension; UEFM: Upper Extremity Fugl-Meyer; CES-D: Center for Epidemiological Studies Depression Scale; MOCA: Montreal Cognitive Assessment; NIHSS: National Institute of Health Stroke Scale

**Fig. 2:**
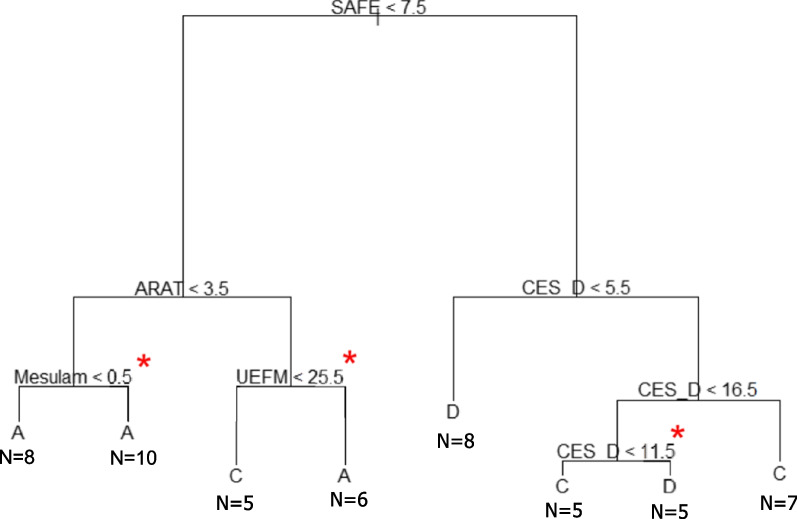
Single unpruned decision tree

The single, unpruned decision tree (Fig. 2) allocated participants into only three of the five UL performance categories. The predictors that were selected for this tree included all three UL clinical measures (SAFE, ARAT, and UEFM) and two non-motor clinical measures (CES-D and Mesulam). This tree has a misclassification rate of 29%, meaning that 16/54 people were misclassified into a different category than their actual. In this tree, the SAFE score is the root node. For participants with less overall strength in their paretic UL (SAFE < 7.5), the left side of the tree is used, with the ARAT, Mesulam, and UEFM scores used to assign people into either category A (Minimal activity/rare integration) or C (Moderate activity/moderate integration). For participants with more overall strength in their paretic UL (SAFE > 7.5), the right side of the tree is used, with participants assigned to either category C (Moderate activity/moderate integration) or D (Moderate activity/full integration) based on their scores on the depression scale (CES-D).

Our next step was to explore the use of bagging methods to build the six models (Table 2). The statistics used to evaluate model performance of the single decision tree, bagged models, and random forest models are presented in Table 4 and the confusion matrices for the models in Additional file 2: Tables S2A–2H. The in-sample accuracy of all the models is better than chance (chance = 0.20 for each of the five categories) alone. The single decision tree has an in-sample accuracy of 0.70 whereas the in-sample accuracy of the bagged and random forest models ranges from 0.96 to 1.00, indicating better performance. The predictive power of the six models was better than chance, with the medium and large models being better than the small models and having mostly overlapping 95% confident intervals.

**Table 4:** Model performance of models built with different machine learning algorithms

	Model name						
Model Performance Statistic	Single Decision Tree	Small		Medium		Large	
		Bagged model	Random forest	Bagged model	Random forest	Bagged model	Random forests
In-sample							
Accuracy^*^							
Mean	0.70	0.96	0.96	1.0	1.0	1.0	1.0
IQR	(0.56, 0.82)	(0.87, 1.0)	(0.87, 0.99)	(0.93, 1.0)	(0.93, 1.0)	(0.93, 1.0)	(0.93, 1.0)
Out-of-bag estimate of error^^^							
Mean 95% CI	na	0.55 (0.54,0.56)	0.52 (0.52,0.54	0.47 (0.46, 0.48)	0.46 (0.44, 0.46)	0.48 (0.46, 0.48)	0.48 (0.48,0.48)

*In-sample accuracy is a measure of the explanatory power of the model and was quantified for all seven models by comparing the predicted category and the actual UL performance categories. Values closer to 1.00 indicate better model performance

^^^Out-of-bag estimate of error is a measure of the predictive power of the models and was quantified for the six bagging models as cross-validation accuracy. Lower error-rate values indicate better model performance

CI: confidence interval; IQR: inter-quartile range

**Fig. 3:**
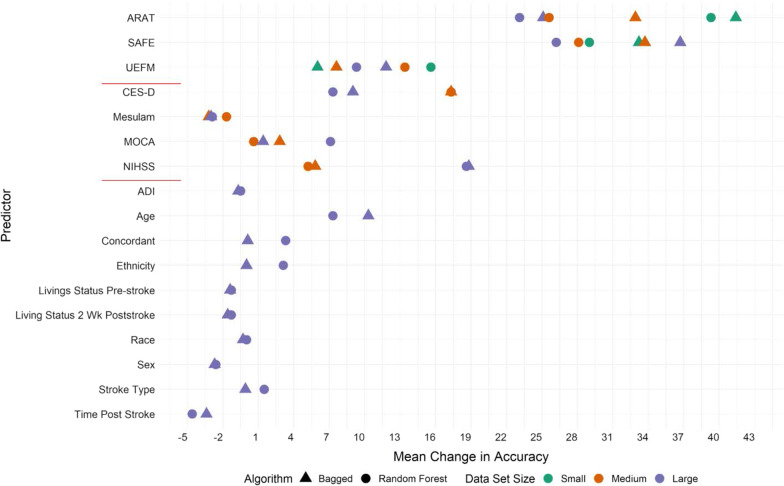
Variable importance plot for the six models built with bagging algorithms from different input datasets and tuning parameters. Variable importance here is computed using the mean change in accuracy (x-axis), and is expressed relative to the maximum. Higher values indicate greater importance of the specific predictor in the model and values < 0 indicate these predictors decrease the overall accuracy of the model. The shape represents the algorithm used and color represents the size of the input dataset. The small data set that includes UL clinical measures, the medium sized data set includes UL clinical measures + non-motor clinical measures, and the large sized data set includes UL clinical measures + non-motor clinical measures + demographics. The bagged models were built with all predictors available in the data set and random forests were built with the square root of the number of predictors

**Fig. 4:**
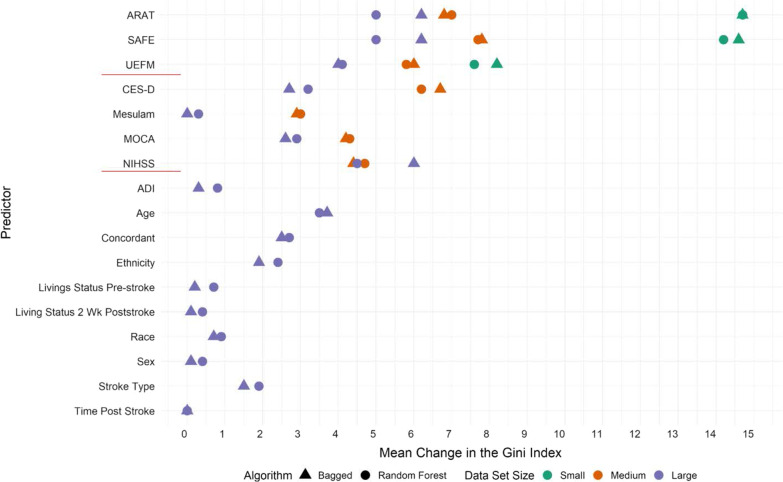
Variable importance plot for the six models built with different input datasets and tuning parameters. Variable importance here is computed using the mean change in the Gini index (x-axis), and is expressed relative to the maximum. The shape represents the algorithm used and color represents the size of the input dataset. The small data set that includes UL clinical measures, the medium sized data set includes UL clinical measures + non-motor clinical measures, and the large sized data set includes UL clinical measures + non-motor clinical measures + demographics. The bagged models were built with all predictors available in the data set and random forests were built with the square root of the number of predictors

Figures 3 and 4 present the variable importance plots for the six models using the mean change in accuracy (Fig. 3) and the mean change in the Gini index (Fig. 4). The two red lines on the y-axis are placed to separate the predictors relative to the data sets with UL clinical measures first, non-motor clinical measures second, and the demographics last. In these plots, shape represents the algorithm, which was either bagged trees (triangle) or random forests (circle) and the three colors represent the size of the input data set as: small (green), medium (orange), or large (purple). In Fig. 3, one can see that two UL clinical measures (ARAT and SAFE) are the most important predictors regardless of the algorithm used or input data set size. Additionally, the UEFM and CES-D emerge as important predictors to maintain accuracy of the models, specifically with the medium and large input data sets. Only one demographic predictor (age) emerged as an important to maintain the accuracy of the models. Interestingly, a few of the demographic predictors (sex, living status pre-stroke, living status 2-weeks post stroke, and time post stroke) all have negative values indicating including these predictors decreases the accuracy of the models. In Fig. 4, the UL clinical measures and non-motor clinical measures are most important with respect to the mean change in the Gini index regardless of the algorithm or input data set size. All UL clinical measures (ARAT, SAFE, and UEFM) have the highest values of the mean change in the Gini index for the small data set only compared to the models built with the medium and large data sets. For most of the predictors, the circles and triangles of the same color are close together indicating similar values for mean change in the Gini index regardless of if the bagged or random forest algorithm was used. Similar to the mean change in accuracy, the only demographic predictor that could be considered important to the models was participant age. Additionally, some of the demographic predictors have a mean change in the Gini index close to 0 indicating less importance of these predictors similar to the mean change in accuracy (Fig. 4).

## Discussion

The purpose of this study was to explore how different machine learning techniques could be used to understand the association between clinical measures and participant demographics captured early after stroke and the subsequent UL performance category. Our hypothesis was supported, such that measures of UL impairment and capacity were the most important predictors of subsequent UL performance category, regardless of the machine learning algorithm used. Other non-motor clinical measures emerged as key predictors, while participant demographic predictors (except for age) were less important across the models. Models built with the bagging algorithms had better in-sample accuracy compared to the single decision tree. The models had moderate out-of-bag errors, indicating that there are likely unmeasured, missing predictive factors. There are two novel contributions of the present study: 1) different machine learning techniques were used to allow for comparison of the results and 2) the outcome of the models was a multi-dimensional category of UL performance. These findings contribute to the understanding of what factors early after stroke may partially influence the subsequent UL performance categories.

Using different machine learning techniques provided information about which predictors were most important to the outcome for this sample and those that generalize to the population. Consistent with efforts to predict an individual’s subsequent UL impairment or UL capacity category [19, 20, 62–65], these results point to the importance of measures of UL impairment and capacity for predicting subsequent UL performance category. The UL impairment (SAFE score, UEFM) and capacity (ARAT) measures were generally the most important predictors regardless of the algorithm or input data set used. Depressive symptomology (CES-D) and overall stroke severity (NIHSS) increased the overall predictive ability of the models. The NIHSS is a measure of global stroke severity, and it is possible that the non-motor aspects of this measure are driving the added value of this predictor. Age was the only demographic predictor that emerged as important across the models, which was not entirely surprising given age’s importance to predicting UL capacity after stroke as well [19, 20]. Collectively these results indicate that the subsequent UL performance categories were most influenced by UL impairment, capacity, presence of depression, overall stroke severity, and age. These are different secondary predictors than were identified in a previous study predicting a single UL performance variable, where non-motor clinical measures of hemispatial neglect and cognitive impairments along with participant demographic information were found to be important [25, 66]. It is reasonable that different factors emerged as predictors of a single UL performance variable vs. a multivariate category of UL performance. A key finding in the current analysis is the substantial out-of-bag estimate of error (0.48 to 0.55), indicating that there are other factors (predictors) that likely contribute to UL performance that were not assessed in the cohort studied here. Other possible factors that could influence UL performance include: biopsychosocial, cognitive constructs (e.g. apraxia), neurobiology (e.g. motor evoked potential [MEP]), and other demographics (e.g. employment status). Biopsychosocial factors (e.g. balance self-efficacy) [35, 37, 67, 68], and demographics (e.g. working vs retired or unemployed and physical environment) [36, 69, 70] have been shown to influence walking performance after stroke where cognitive constructs (e.g. apraxia) [32, 71] and social and emotional factors (e.g. intrinsic motivation and self-determination) influence UL performance after stroke [72, 73]. Neurobiological markers, such as presence of an MEP, improve the prediction of UL capacity outcomes for people with less initial strength in their paretic UL [19, 20]. Future research should explore how these factors captured early after stroke are associated with subsequent UL performance.

The purpose of the present analysis was not to make perfect predictions, but rather to explore the associations between these variables using different machine learning techniques. The first model we explored was a single decision tree built with the CART algorithm. While the graphical representation of this decision tree (Fig. 2) may be easy to interpret, the output is somewhat counterintuitive. For example, on the left side of the tree for people with a SAFE score < 7.5 and an ARAT score > 3.5, it is counterintuitive that a UEFM score < 25.5 (more UL impairment) would put one in a better category (C, Moderate activity/moderate integration), and an UEFM > 25.5 would put one in a worse category (A, Minimal activity/rare integration). This likely occurred because the tree was constructed with all the input data and the algorithm assigned people to the categories based on the probability of ending up in each node. It is possible that there were a few people in this data set that had this unusual pattern of scores with respect to UL impairment and capacity. Likewise, the tree selected the Mesulam, a non-motor clinical measure of hemispatial neglect, for the most leftward node, but inclusion of this node does not change the categorization. Then, two different bagging algorithms were used to build predictive models with different sized input data sets to explore if they yielded similar versus different results. A benefit of the bagged and random forest algorithms was the relative increase in the explanatory and predictive power of these models because of cross-validation, even with the smaller sample size of our data set, as seen in Table 4. The in-sample accuracy for these six models was improved compared to the single decision tree, indicating that these models are doing a good job explaining the data we had. The out-of-bag estimate of error, however, is more important with respect to the predictive power of these models. While the out-of-bag estimate of error does decrease (indicating more accurate predictions) with the addition of the non-motor clinical measures, the outcome of the models remained largely unchanged, and the out-of-bag error remains substantial. These data illustrate the point that, ultimately, prediction models may only be as “good” as the input data available; simply switching to a different machine learning method with different tuning parameters may not substantially change the predictive ability of the model. While we did not try all possible machine learning algorithms (e.g. SVM or neural networks), this could be an important consideration for future research. The single decision tree is a transparent model because one can see how the decisions are made in the tree where the bagged and random forest models are harder to interpret, because the classification is based on thousands of trees. These ensemble classifiers, however, can still be clinically useful. As we move to a world where electronic health records are integrated into advanced information management systems with data visualization and machine learning capability [74], the possibilities are endless to imagine how clinical measures and participant demographics early after stroke could be used to predict meaningful outcomes for people with stroke and their families. Implementation of these techniques into routine care will require extremely large data sets to build and then to validate models. Sample sizes will need to be at least an order of magnitude bigger than the larger data sets available today (i.e. in the thousands, not tens or hundreds of participants) [28, 29]. For these machine learning methods to be clinically-available in the future, research groups need to start pooling participant data, data sharing, and/or using more common data elements across studies.

There are a few limitations to consider when interpreting our findings. First, these results should be interpreted as exploratory or “hypothesis-generating”. Additional studies are required to validate these results. Second, due the small sample size the data could not be split into test and training sets. Nonetheless, we were still able to capitalize on the computing power of these techniques to provide additional information that contributes to our understanding of how typical information captured early after stroke was associated with a subsequent UL performance category. Third, categories B: Minimal activity/limited integration and E: High activity/full integration are under-represented in this sample. We purposefully chose not to resample from these categories to create more balanced groups because of the potential effect on the bias-variance trade-off in the models [61]. However, both the in-sample and out-of-bag accuracy values were higher than the no information rate (0.30) for all of the seven models. An important goal of future work will be to ensure more equal representation across the categories with a larger sample size. Finally, the predictor sets only included clinical measures and participant demographic information because we were limited by the data collected for the prospective, observational, longitudinal cohort study and the variability of the predictors across the cohort [32]. One example of a potential predictor variable not collected here is a positive motor evoked potential (MEP), which has been identified as an important factor predicting UL capacity for persons with greater UL impairment in their paretic UL [19, 20, 25]. As an example of lack of variability in potential predictors, we also collected a survey quantifying self-perception of UL performance recovery. Scores on these measures were highly homogenous across participants, making it impossible for this factor to contribute to the variance in the outcome. Future studies will need to be designed with a more comprehensive set of potential predictors, including neurobiological and psychosocial factors.

## Conclusion

Machine learning techniques can be used to understand how clinical measures and participant demographics captured early are associated with subsequent post stroke UL performance categories. UL clinical measures were the most important predictors of the subsequent UL performance category in this exploratory analysis regardless of the machine learning algorithm used. Other non-motor clinical measures emerged as important predictors to maintain the accuracy of the models, but including these measures had little impact on the out-of-bag error. These results reinforce that UL performance, in vivo, is not a simple product of body functions nor the capacity for UL movement, instead being a complex phenomenon dependent on many physiological and psychological factors. Utilizing machine learning, this exploratory analysis is a productive step towards prediction of UL performance. Future research is required to explore other factors associated with UL performance along with the role of predictive models in rehabilitation after stroke.

## Supplementary Information

**Additional file 1: Figure S1.** Correlational matrix and coefficients of the included continuous predictors.

**Additional file 2: Table S1.** Frequency of responses for the five demographic variables excluded in this analysis as potential predictors. **Table S2A.** Confusion matrix of baseline categories to actual categories. **Table S2B.** Confusion matrix of single unpruned decision tree. **Table S2C.** Confusion matrix of small bagged model. **Table S2D.** Confusion matrix of small random forest model. **Table S2E.** Confusion matrix of medium bagged model. **Table S2F.** Confusion matrix of medium random forest model. **Table S2G.** Confusion matrix of large bagged model. **Table S2H.** Confusion matrix of large random forest model.

## Data Availability

The data analyzed in the present study are in the process of being added to available at the NICHD/NCMRR Restore Center, https://restore.stanford.edu/. The Center provides open-access data via their SimTK platform: https://simtk.org/projects/referentaccdata.
