# Supplementary figures and images for: Predicting later categories of upper limb activity from earlier clinical assessments following stroke: an exploratory analysis
Source: J Neuroeng Rehabil. 2023 Feb 21;20:24. doi: 10.1186/s12984-023-01148-1 (PMC9945671; doi:10.1186/s12984-023-01148-1)

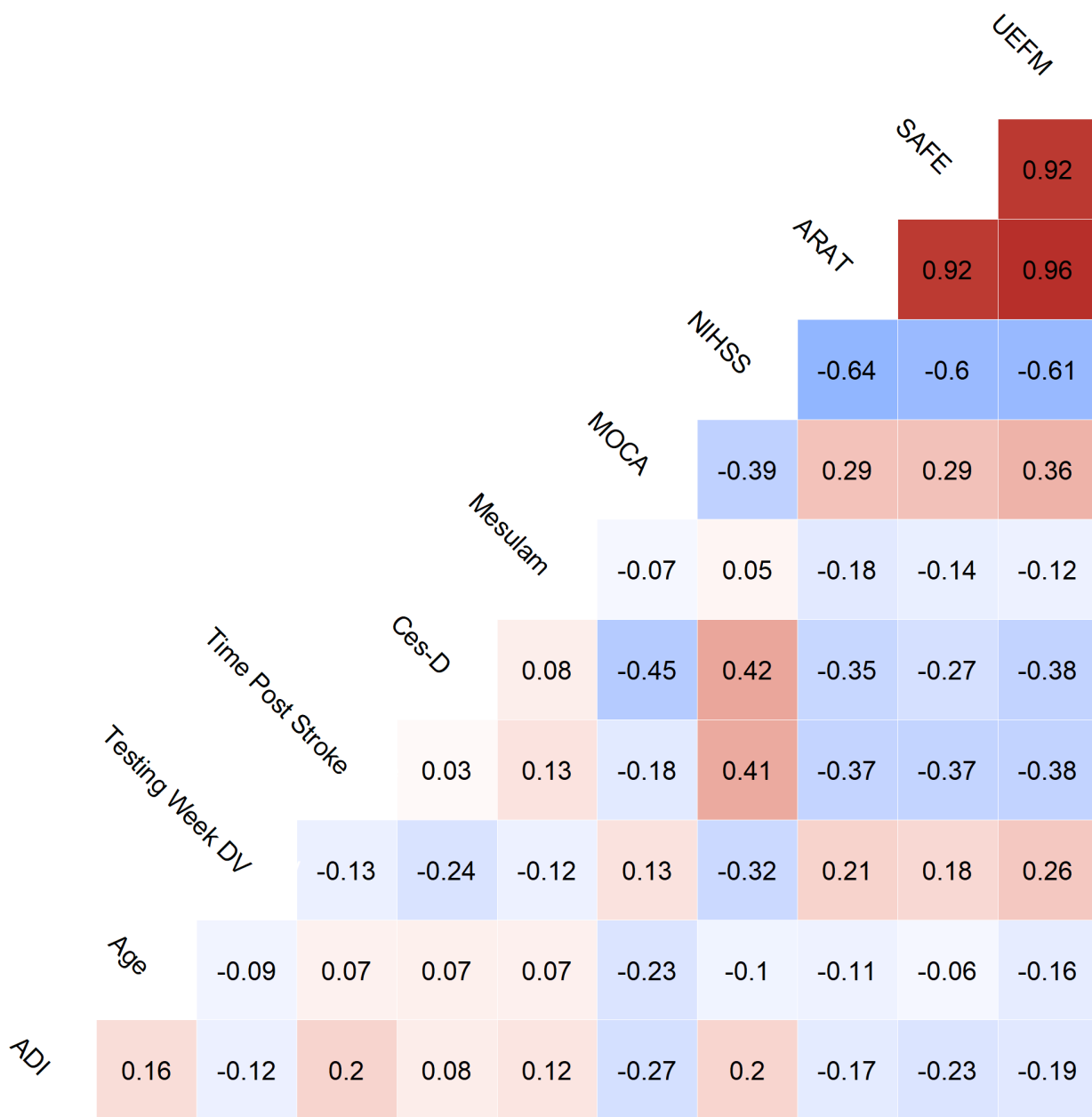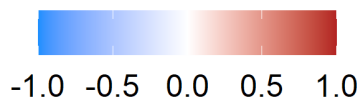

Supplement: Supplementary file 1 — Additional file 1: Figure S1. Correlational matrix and coefficients of the included continuous predictors. [file 12984_2023_1148_MOESM1_ESM.pdf]
