# Supplementary material for: Predicting later categories of upper limb activity from earlier clinical assessments following stroke: an exploratory analysis
Source: J Neuroeng Rehabil. 2023 Feb 21;20:24. doi: 10.1186/s12984-023-01148-1 (PMC9945671; doi:10.1186/s12984-023-01148-1)
Supplement: Supplementary file 2 — Additional file 2: Table S1. Frequency of responses for the five demographic variables excluded in this analysis as potential predictors. Table S2A. Confusion matrix of baseline categories to actual categories. Table S2B. Confusion matrix of single unpruned decision tree. Table S2C. Confusion matrix of small bagged model. Table S2D. Confusion matrix of small random forest model. Table S2E. Confusion matrix of medium bagged model. Table S2F. Confusion matrix of medium random forest model. Table S2G. Confusion matrix of large bagged model. Table S2H. Confusion matrix of large random forest model. [file 12984_2023_1148_MOESM2_ESM.docx]

**Figure S1.** Correlational matrix and coefficients of the included continuous predictors.

Table S1: Frequency of responses for the five demographic variables excluded in this analysis as potential predictors.

| Categorical Demographic Variables | Total Sample  N=54 |
| --- | --- |
| Working prior, n (%)  Yes  No | 20 (37)  34 (63) |
| Levels of employment, **^*^** n (%)  Not paid  Less than 20 hours per week  More than hours per week  Full time | 0 (0)  2 (10)  3 (15)  15 (75) |
| Education, n (%)  8^th^ grade  High school  Vocational/Tech School  Associates degree  Some college  Bachelor’s degree  Masters, doctoral, professional degree | 1 (2)  21 (39)  5 (9)  1 (2)  17 (31)  3 (6)  5 (10) |
| Comorbidity,**^Ւ^** n (%)  Arthritis  Bleeding problems  Cancer  Diabetes  Heart problems  Hepatitis  High blood pressure  Infections  Kidney problems  Liver problems  Lung problems  Mental health  Muscle/bone problems  Neurological problems  Seizures  Stomach problems  Stroke  Thyroid problems  Vision problems  Other  None | 13 (24)  1 (2)  10 (5)  19 (35)  20 (37)  2 (4)  39 (72)  1 (2)  2 (4)  3 (6)  3 (6)  1 (2)  0  0  0  3 (6)  2 (39)  5 (9)  4 (7)  7 (13)  2 (4) |

* Levels of employment were only captured for those who were working prior to the stroke. % is calculated out of 20.

**^Ւ^** Comorbidities presented are the number of people who reported that they had the condition.

Table S2A. Confusion matrix of baseline categories to actual categories.

|  | Final Category | | | | | |
| --- | --- | --- | --- | --- | --- | --- |
| Baseline  Category | A | B | C | D | E | SUM |
| A | 20 (37%) | 2 (4%) | 4 (7%) | 1 (2%) | 0 | 27 (50%) |
| B | 0 | 1 (2%) | 5 (9%) | 2 (4%) | 2 (4%) | 10 (19%) |
| C | 0 | 1 (2%) | 6 (11%) | 5 (9%) | 1 (2%) | 13 (24%) |
| D | 0 | 0 | 1 (2%) | 2 (4%) | 1 (2%) | 4 (7%) |
| E | 0 | 0 | 0 | 0 | 0 | 0 |
| SUM | 20 (37%) | 4 (7%) | 16 (30%) | 10 (19%) | 4 (7%) | 54 |

Supplemental Table 2B. Confusion matrix of single Unpruned decision tree

|  | Actual Category | | | | | |
| --- | --- | --- | --- | --- | --- | --- |
| Predicted  Category | A | B | C | D | E | SUM |
| A | 19 (35%) | 2 (3%) | 1 (2%) | 2 (3%) | 0 | 24 (44%) |
| B | 0 | 0 | 0 | 0 | 0 | 0 |
| C | 1 (2%) | 2 (3%) | 12 (22%) | 1 (2%) | 1 (2%) | 17 (32%) |
| D | 0 | 0 | 3 (6%) | 7 (13%) | 3 (6%) | 13 (24%) |
| E | 0 | 0 | 0 | 0 | 0 | 0 |
| SUM | 20 (37%) | 4 (7%) | 16 (30%) | 10 (19%) | 4 (7%) | 54 |

Table S2C. Confusion matrix of small bagged model

|  | Actual Category | | | | | |
| --- | --- | --- | --- | --- | --- | --- |
| Predicted  Category | A | B | C | D | E | SUM |
| A | 20 (37%) | 2 (4%) | 0 | 0 | 0 | 22 (41%) |
| B | 0 | 2 (4%) | 0 | 0 | 0 | 2 (4%) |
| C | 0 | 0 | 16 (30%) | 0 | 0 | 16 (30%) |
| D | 0 | 0 | 0 | 10 (19%) | 0 | 10 (19%) |
| E | 0 | 0 | 0 | 0 | 4 (7%) | 4 (7%) |
| SUM | 20 (37%) | 4 (7%) | 16 (30%) | 10 (19%) | 4 (7%) | 54 |

Table S2D. Confusion matrix of small random forest model

|  | Actual Category | | | | | |
| --- | --- | --- | --- | --- | --- | --- |
| Predicted  Category | A | B | C | D | E | SUM |
| A | 20 (37%) | 2 (4%) | 0 | 0 | 0 | 22 (41%) |
| B | 0 | 2 (4%) | 0 | 0 | 0 | 2 (4%) |
| C | 0 | 0 | 16 (30%) | 0 | 0 | 16 (30%) |
| D | 0 | 0 | 0 | 10 (19%) | 0 | 10 (19%) |
| E | 0 | 0 | 0 | 0 | 4 (7%) | 4 (7%) |
| SUM | 20 (37%) | 4 (7%) | 16 (30%) | 10 (19%) | 0 (7%) | 54 |

Table S2E. Confusion matrix of medium bagged model

|  | Actual Category | | | | | |
| --- | --- | --- | --- | --- | --- | --- |
| Predicted  Category | A | B | C | D | E | SUM |
| A | 20 (37%) | 0 | 0 | 0 | 0 | 20 (37%) |
| B | 0 | 4 (7%) | 0 | 0 | 0 | 4 (7%) |
| C | 0 | 0 | 16 (30%) | 0 | 0 | 16 (30%) |
| D | 0 | 0 | 0 | 10 (19%) | 0 | 10 (19%) |
| E | 0 | 0 | 0 | 0 | 4 (7%) | 4 (7%) |
| SUM | 20 (37%) | 4 (7%) | 16 (30%) | 10 | 4 (7%) | 54 |

Table S2F. Confusion matrix of medium random forest model

|  | Actual Category | | | | | |
| --- | --- | --- | --- | --- | --- | --- |
| Predicted  Category | A | B | C | D | E | SUM |
| A | 20 (37%) | 0 | 0 | 0 | 0 | 20 (37%) |
| B | 0 | 4 (7%) | 0 | 0 | 0 | 4 (7%) |
| C | 0 | 0 | 16 (30%) | 0 | 0 | 16 (30%) |
| D | 0 | 0 | 0 | 10 (19%) | 0 | 10 (19%) |
| E | 0 | 0 | 0 | 0 | 4 (7%) | 4 (7%) |
| SUM | 20 (37%) | 4 (7%) | 16 (30%) | 10 (19%) | 4 (7%) | 54 |

Table 2G. Confusion matrix of large bagged model

|  | Actual Category | | | | | |
| --- | --- | --- | --- | --- | --- | --- |
| Predicted  Category | A | B | C | D | E | SUM |
| A | 20 (37%) | 0 | 0 | 0 | 0 | 20 (37%) |
| B | 0 | 4 (7%) | 0 | 0 | 0 | 4 (7%) |
| C | 0 | 0 | 16 (30%) | 0 | 0 | 16 (30%) |
| D | 0 | 0 | 0 | 10 (19%) | 0 | 10 (19%) |
| E | 0 | 0 | 0 | 0 | 4 (7%) | 4 (7%) |
| SUM | 20 (37%) | 4 (7%) | 16 (30%) | 10 (19%) | 4 (7%) | 54 |

Table S2H. Confusion matrix of large random forest model.

|  | Actual Category | | | | | |
| --- | --- | --- | --- | --- | --- | --- |
| Predicted  Category | A | B | C | D | E | SUM |
| A | 20 (37%) | 0 | 0 | 0 | 0 | 20 (37%) |
| B | 0 | 4 (7%) | 0 | 0 | 0 | 4 (7%) |
| C | 0 | 0 | 16 (30%) | 0 | 0 | 16 (30%) |
| D | 0 | 0 | 0 | 10 (19%) | 0 | 10 (19%) |
| E | 0 | 0 | 0 | 0 | 4 (7%) | 4 (7%) |
| SUM | 20 (37%) | 4 (7%) | 16 (30%) | 10 (19%) | 4 (7%) | 54 |
